# Space-type radiation induces multimodal responses in the mouse gut microbiome and metabolome

**DOI:** 10.1186/s40168-017-0325-z

**Published:** 2017-08-18

**Authors:** David Casero, Kirandeep Gill, Vijayalakshmi Sridharan, Igor Koturbash, Gregory Nelson, Martin Hauer-Jensen, Marjan Boerma, Jonathan Braun, Amrita K. Cheema

**Affiliations:** 1 Department of Pathology and Laboratory Medicine, David Geffen School of Medicine, University of California, Los Angeles, Los Angeles, CA 90095 USA; 2 Department of Oncology, Georgetown University Medical Center, Washington DC, 20057 USA; 3 Division of Radiation Health, University of Arkansas for Medical Sciences, Little Rock, AR 72205 USA; 4 Department of Environmental and Occupational Health, University of Arkansas for Medical Sciences, Little Rock, AR 72205 USA; 5 Department of Radiation Medicine, Loma Linda University, Loma Linda, CA 92350 USA; 6 Department of Biochemistry and Molecular and & Cellular Biology, Georgetown University Medical Center, Washington, DC 20057 USA; 7 GCD-7N Pre-Clinical Science Building, 3900 Reservoir Road NW, Washington DC, 20057 USA

**Keywords:** Ionizing radiation, Space travel, Microbiome, 16S rRNA amplicon sequencing, Untargeted metabolomics, Metabolic network modeling

## Abstract

**Background:**

Space travel is associated with continuous low dose rate exposure to high *linear energy transfer* (*LET*) radiation. Pathophysiological manifestations after low dose radiation exposure are strongly influenced by non-cytocidal radiation effects, including changes in the microbiome and host gene expression. Although the importance of the gut microbiome in the maintenance of human health is well established, little is known about the role of radiation in altering the microbiome during deep-space travel.

**Results:**

Using a mouse model for exposure to high LET radiation, we observed substantial changes in the composition and functional potential of the gut microbiome. These were accompanied by changes in the abundance of multiple metabolites, which were related to the enzymatic activity of the predicted metagenome by means of metabolic network modeling. There was a complex dynamic in microbial and metabolic composition at different radiation doses, suggestive of transient, dose-dependent interactions between microbial ecology and signals from the host’s cellular damage repair processes. The observed radiation-induced changes in microbiota diversity and composition were analyzed at the functional level. A constitutive change in activity was found for several pathways dominated by microbiome-specific enzymatic reactions like carbohydrate digestion and absorption and lipopolysaccharide biosynthesis, while the activity in other radiation-responsive pathways like phosphatidylinositol signaling could be linked to dose-dependent changes in the abundance of specific taxa.

**Conclusions:**

The implication of microbiome-mediated pathophysiology after low dose ionizing radiation may be an unappreciated biologic hazard of space travel and deserves experimental validation. This study provides a conceptual and analytical basis of further investigations to increase our understanding of the chronic effects of space radiation on human health, and points to potential new targets for intervention in adverse radiation effects.

**Electronic supplementary material:**

The online version of this article (doi:10.1186/s40168-017-0325-z) contains supplementary material, which is available to authorized users.

## Background

In the context of ongoing programs for human exploration mission to Mars and deep space, there is an emerging interest in how the microbiome may predispose an individual to radiation injury and how radiation-induced modifications in the microbiome affect the individual’s overall response to radiation [1, 2]. Together with microgravity and other environmental factors in space, ionizing radiation is a likely contributor to alterations in the microbiome. The gut microbiome has evolved as a symbiotic ecosystem that contributes specific and essential biochemical reactions to its host [3]. One can therefore anticipate that protracted low dose exposures to radiation can potentially induce long-term alterations in gut homeostasis; however, radiation-induced alterations along the host-microbiome axis associated with health risks have not been fully characterized. Although the importance of the microbiome in the maintenance of human health during space travel has been recognized [4, 5], little is known about the role of radiation in altering the microbiome during deep-space travel [6–8]. Previously, we have shown that exposure to heavy ions (^56^Fe) causes oxidative stress and dysregulated prostanoid biosynthesis in the mouse intestinal metabolome [9]. However, the correlation and the impact of the microbiota remained to be elucidated.

Space travel beyond the low Earth orbit is associated with the risk of exposure to high linear energy transfer (LET) ionizing radiation, mainly due to galactic cosmic rays (GCR), solar emissions, and solar particle events (SPEs). SPEs are predominantly associated with high dose rate exposures to protons, while GCR include iron, silicon, oxygen, carbon, and helium ions that are highly energetic and cannot be easily shielded by practical levels of existing shielding materials used during space travel. The chronic radiation exposure from GCR, when outside the protective environment of the earth’s magnetosphere, occurs at a dose rate of 1.3 mGy/day, and total doses of a return mission to Mars can add up to 0.5 Gy [10, 11]. While there are concerns about the systemic effects of exposure to space radiation [12, 13], long-term degenerative tissue and organ effects of chronic exposures to GCR have not been characterized [14]. As such, more research is needed for the identification of specific changes that underscore short- and long-term health risks of exposure to high LET radiation, in conditions that space travelers are likely to encounter in deep space.

**Fig. 1:**
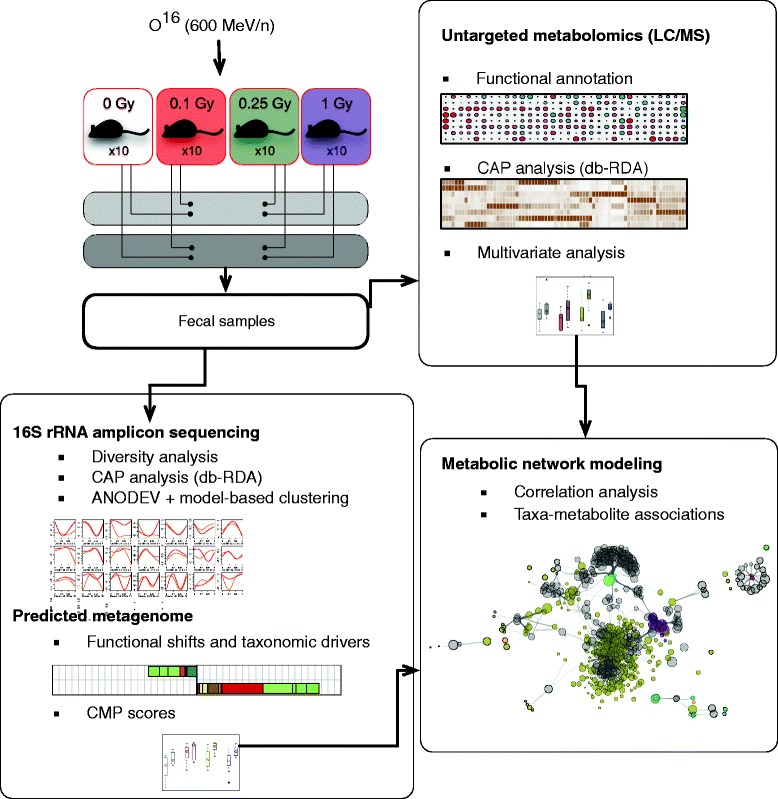
Experimental and analytical design. Fecal samples were collected from irradiated mice and processed for both 16S rRNA amplicon and LC-MS profiling. 16S rRNA amplicon data was analyzed at the phylotype level unless stated otherwise. Constrained Analysis of Principal Coordinates (CAP) provided condition-specific phylotypes and metabolites, while model-based clustering produced a classification of highly responsive phylotypes based on overall response to irradiation. The predicted metagenome was employed to estimate contributions of bacterial phylotypes to significant functional shifts and community-wide metabolic potential (CMP) scores. Metabolic network modeling was used to integrate the 16S rRNA amplicon and metabolomics data and to establish significant associations between phylotypes and metabolic shifts

Herein, we used 16S rRNA amplicon sequencing, untargeted metabolomics, and metabolic network modeling (Fig. 1) to produce a multi-omics narrative of intestinal metabolism in a mouse model of (heavy ion) extra-terrestrial irradiation exposure (^16^O). We report a complex dynamics of the gut ecosystem post-radiation, with time-modulated abundances for both commensal and opportunistic microbial species. Concomitant with these changes, we observed a shift on the abundance of multiple metabolites, which could contribute to the onset and progression of radiation-induced disorders in a dose- and time-dependent manner. Metabolic network modeling suggested that the inferred metagenome is a good predictor of the observed metabolic state. Finally, we found a dose-dependent response to radiation in the microbiome, with increased sensitivity at lower doses (0.1 and 0.25 Gy). This threshold-like behavior is suggestive of a complex host-microbiome interaction in response to radiation that might result from signals involved in DNA damage and cell survival. This work provides a framework to identify host-microbiome responses that might elevate health risks after exposure to space-type ionizing radiation.

## Results

### Changes in the fecal microbiome of mice exposed to low dose high LET radiation

**Fig. 2:**
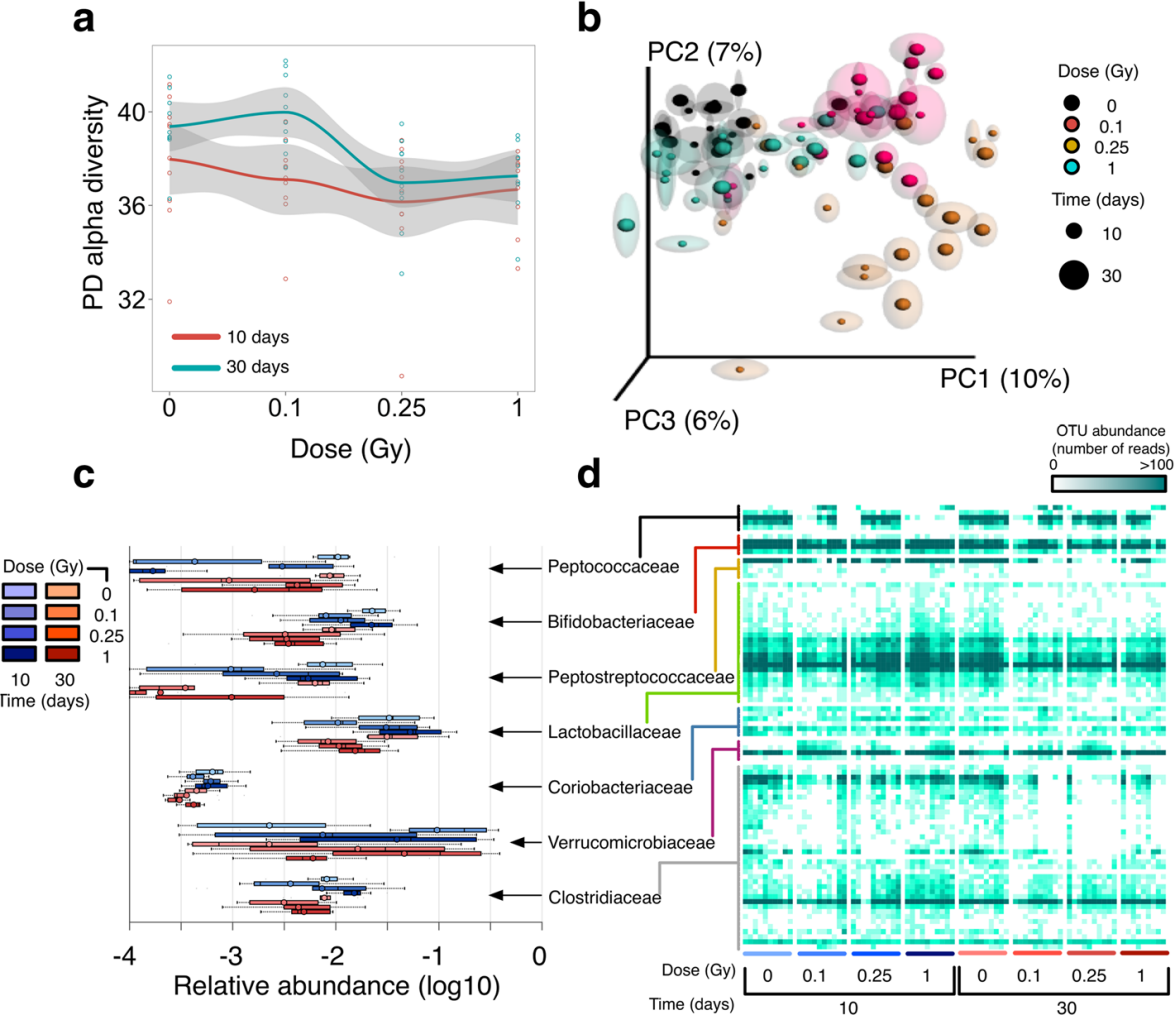
Ecological analysis of the irradiated microbiome. **a** Alpha diversity for control and irradiated samples 10 (*red*) and 30 (*blue*) days post-radiation. Shown are per-sample (*dots*), and per-condition averages (*line plots*), and standard deviations (*gray bands*). Values correspond to Faith’s phylogenetic diversity metric (PD). **b** Jackknifed Principal Coordinate Analysis (PCoA) plot of UniFrac unweighted distances between sample groups. For each sample, shown are confidence ellipses obtained from independent random rarefactions of the OTU counts table. **c** Barplots of per-condition relative abundances (logarithmic scale) for bacterial families with significant variations across conditions (Bonferroni *p* value < 0.05, Kruskal-Wallis test). **d** Heatmap of phylotype-level counts. All samples (*columns*) are shown and grouped by experimental factors. Individual phylotypes (*rows*) are grouped at the family level

We started by asking if the composition of the fecal microbiota was modulated by exposure to charged particle radiation. We collected fecal samples from mice after 10 and 30 days of exposure to ^16^O (600 MeV/n) at 0.1, 0.25, and 1 Gy or sham-treatment (non-irradiated mice; Fig. 1). Bacterial composition was inferred from the analysis of 16S rRNA amplicon sequencing data. Diversity analysis revealed an intricate relationship between bacterial richness and radiation dose. Overall, mice subjected to radiation showed a slight decrease in bacterial diversity (Fig. 2a) as compared to non-irradiated controls. Moreover, alpha diversity was significantly different (*p* value < 0.006; nonparametric *t* test of phylogenetic diversity) between the 10- and 30-day samples, mostly due to a marked increase in diversity at 30 days for mice radiated at 0.1 Gy (Fig. 2a).

Multivariate analysis to determine the effect of the experimental factors on bacterial composition (beta diversity analysis) showed that both *time after exposure* (*Time* hereafter) and *total dose* (*dose* hereafter) have a significant impact on bacterial community structure (*p* values < 0.005 and < 0.001, respectively; ANOSIM nonparametric test on unweighted UniFrac distances, Additional file 1: Table S1). Binary comparisons between fecal samples of irradiated and non-irradiated animals revealed a significant difference in beta diversity regardless of dose levels (*p* value < 0.001; ANOSIM nonparametric test on unweighted UniFrac distances, Additional file 1: Table S1) at both the 10- and 30-day time points. In contrast, no difference was found from pairwise comparisons between groups of mice at the same radiation dose (*p* value > 0.05; ANOSIM nonparametric test on unweighted UniFrac distances, Additional file 1: Table S1). The results from these tests were recapitulated in the PCoA ordination plots of the first three principal components of the unweighted UniFrac distance matrix (Fig. 2b). Strikingly, this ordination shows a pronounced modulation of the composition of the fecal microbiota from mice exposed to 0.1 and 0.25 Gy, while those exposed to a much higher dose (1 Gy) consistently clustered with controls in the PCoA space.

Collectively, our ecological analysis revealed an intricate dose-dependent response to ionizing radiation in the gut microbiome, with enhanced sensitivity for the lowest doses employed here. On the other hand, a distinct reorganization of the microbiota was observed at different doses as soon as 10 days post-radiation. This initial perturbation was followed by a restrained modulation at later times (30 days) without appreciable changes in community structure.

### Phylotype-level dynamics of the microbiota after irradiation

To characterize bacterial homeostasis post-radiation, we next determined the specific taxonomies that were significantly regulated in our samples. As expected, the normal gut microbiota commensals, *Bacteroidetes* (40 and 44%) and *Firmicutes* (56 and 51%) phyla dominated the fecal microbiota of non-irradiated mice at 10 and 30 days (Additional file 2: Table S2). Exposure to low dose high LET radiation was observed to induce significant fluctuations on the prevalence of highly abundant phyla, with a concomitant variation in rare taxa. In fact, group significance analysis showed a significant perturbation on the relative abundance of bacteria in the order of *Bifidobacteriales* and *Coriobacteriales* (*Actinobacteria*), and *Verrucomicrobiales* (*Verrucomicrobia*), along with *Lactobacillales* (*Firmicutes*). Figure 2c shows the relative abundance of bacterial families that tested significant in our factorial design (Bonferroni *p* value < 0.05, Kruskal-Wallis test, Additional file 2: Table S2). The preceding findings were recapitulated from the results of linear discriminant analysis (LDA) effect size (LEfSe) analysis (Additional file 3: Table S3 and Additional file 4: Figure S1). In particular, the relative abundance of *Verrucomicrobia* species increased to prominent levels for specific combinations of dose and time (e.g., up to ~ 18% for 0.1 Gy at 10 days, as compared to < 1% for non-irradiated controls). As a result, LEfSe classified the order of *Verrucomicrobiales* with maximal positive LDA effect size in some cases, suggesting a prominent role of *Verrucomicrobia* in the opportunistic colonization of the mouse gut after exposure to low doses of high LET radiation.

**Fig. 3:**
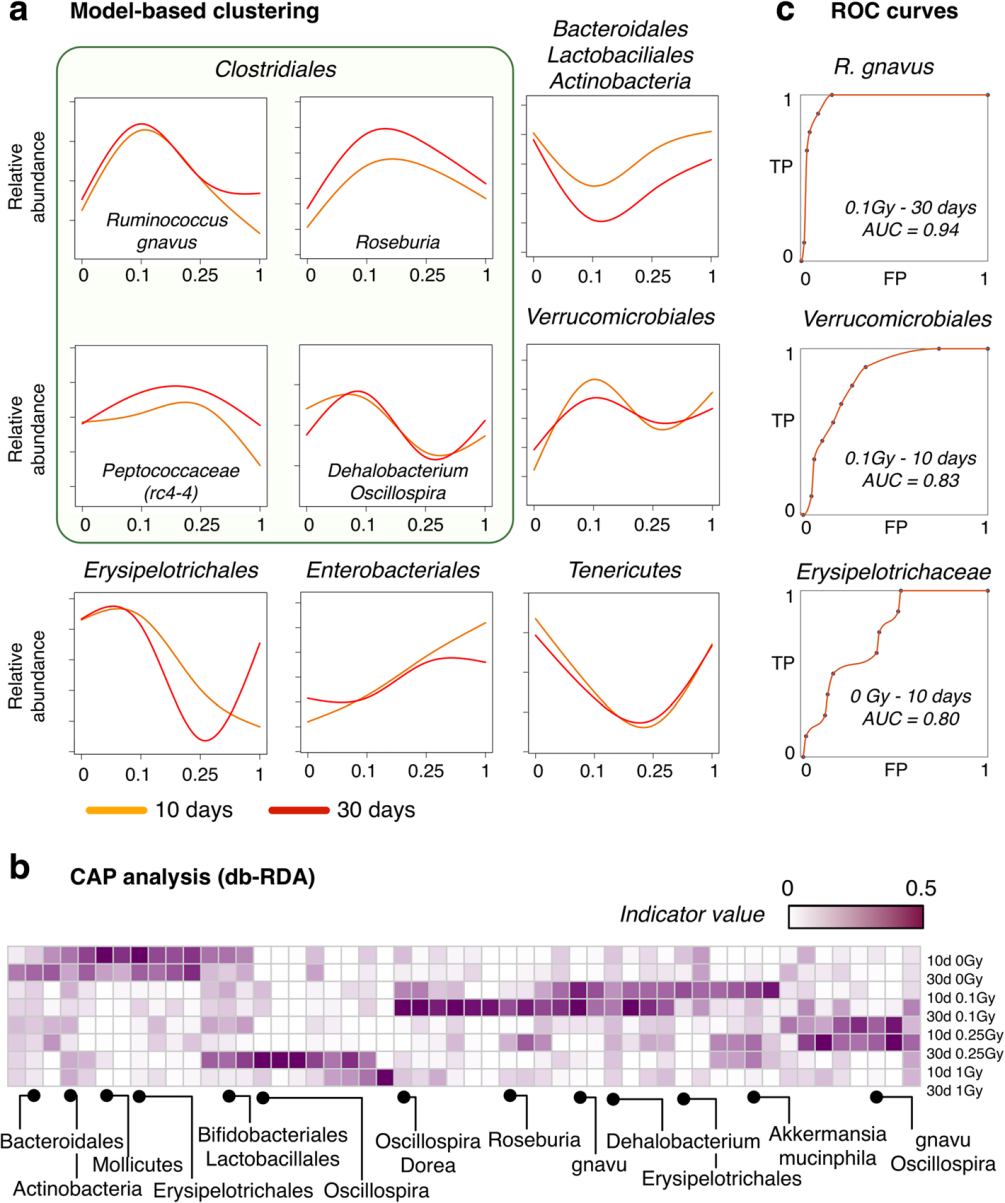
Phylotype-level classification of the irradiated microbiome. **a** Model-based clustering of phylotypes based on overall abundance profiles. Shown are clusters enriched in specific taxonomic groups (hypergeometric *p* value < 0.05). Full results are provided in Additional file 4: Figure S2a. *Line plots* represent the average abundance profile for all phylotypes classified in each cluster. **b** Heatmap of per-group indicator values (distance-based Redundancy Analysis; db-RDA) for selected condition-specific phylotypes. *Labels* represent higher-order taxonomic levels of those phylotypes for greater clarity (*gnavu = Ruminococcus gnavus*). **c** Receiver operating characteristic (ROC) curves for selected conditions and condition-specific taxa. TP = true positive rate, FP = false positive rate, AUC = area under the curve

Although taxonomic changes at the family level reached statistical significance (Fig. 2c; Additional file 2: Table S2), moderate differences for radiated samples from the same group were observed in some cases (Fig. 2d) for both highly abundant and rare phylotypes, which could be due to individual variations in the temporal modulation described above. Therefore, we next aimed to produce a parsimonious, unsupervised classification of phylotypes based on their relative abundance profile. To this end, we fitted our phylotype counts matrix to different models using *Generalized Linear Model* (GLM) fitting (see Methods), which allowed us to identify all OTUs that were affected by our experimental factors (496 OTUs, FDR < 0.01). This pool of candidate phylotypes was then subjected to unsupervised *Model-based clustering* for profile-based classification. Figure 3a highlights the results for those taxa where a significant over-representation of their corresponding phylotypes in specific clusters was found (hypergeometric *p* value < 0.05, see Additional file 4: Figure S2a and Additional file 5: Table S4 for a complete summary).

Bacterial taxa generally considered beneficial were significantly classified in a cluster describing a time-modulated dose response (Fig. 3a; cluster 20 in Additional file 2: Figure S2a). Specifically, *Actinobacteria* (*Bifidobacterium* genus and *Adlercreutzia* unclassified species), *Bacteroidetes* (*S24–7* unclassified species), and *Firmicutes* (unclassified *Lactobacillus* and *Clostridiaceae* species) were observed to decrease their relative abundance in irradiated samples 30 days after exposure as compared to their 10-day counterparts. However, this downturn was observed earlier and to be of greater magnitude for low (0.1 and 0.25 Gy) radiation doses, which again hints to unknown modulatory factors that are activated at high doses.

On the other hand, *Mollicutes* species (*Tenericutes* phylum) were also typically found to extinguish after exposure to 0.25 Gy of ^16^O (clusters 19 and 20, Additional file 2: Figure S2a). Concordant with the previous observations and the group significance analysis described above, a number of *Verrucomicrobia* phylotypes annotated as *Akkermansia muciniphila* were observed to increase their relative abundance. However, a complex interaction between Dose and Time was observed for these phylotypes, which were classified in a cluster showing a strong dose-dependent temporal dynamics (Fig. 3a; cluster 37 in Additional file 4: Figure S2a) with distinct phases of blooming and decline along with a pronounced response to low doses (0.1 Gy).

Remarkably, we observed a heterogeneous array of abundance profiles for phylotypes in the order of *Clostridiales*, a dominant class of gut commensal bacteria*.* For instance, unclassified *Dehalobacterium* (*Dehalobacteriaceae*) and *Oscillospira* (*Ruminococcaceae*) species were mostly classified as phylotypes with fluctuating abundance profile in the Time/Dose space (Fig. 3a; cluster 28 in Additional file 4: Figure S2a). On the other hand, phylotypes classified under the prevalent *Lachnospiraceae* family showed a strong interaction between *Time* and *Dose* across different clusters (Additional file 5: Table S4). Of note, the butyrate-producing *Roseburia* genera showed a marked increase in abundance 30 days after exposure, mostly for 0.1 and 0.25 Gy (Fig. 3a). *Ruminococcus gnavus* showed a marked expansion at 0.1 Gy that persisted or was amplified 30 days after exposure for some phylotypes, while *Peptococcaceae* species including the abundant *rc4–4*, reached normal levels at 30 days after a decline at 10 days in most animals exposed to 0.1 Gy of ^16^O radiation. With respect to the *Firmicutes* phylum, the most abundant species in the *Erysipelotrichaceae* family was classified in cluster 19 (Additional file 4: Figure S2a) with decimated abundance post-radiation, while an unclassified species in the *Allobaculum* genus showed an opportunistic, blooming profile (Fig. 3a; cluster 5 in Additional file 2: Figure S2a).

The foregoing unsupervised classification of the fecal microbiota outlines the response to radiation in the murine gut ecosystem as a function of *Dose* and *Time*. We next aimed to test if, alternatively, the overabundance of a restricted set of phylotypes can segregate specific combinations of Dose and Time*.* To this end, rarefied 16S rRNA counts were subjected to *Constrained Analysis of Principal Coordinates* (*CAP*) by means of the *db-RDA* approach (see Methods and Additional file 6: Table S5). This analysis confirmed that the global ordination of our samples is explained by a diverse array of phylotypes from different taxonomic orders, with *Firmicutes* and *Verrucomicrobiales* as the more significant classifiers (db-RDA *p* value < 0.001), although some *Tenericutes* and *Actinobacteria* (Bifidobacteriaceae and Coriobacteriaceae) phylotypes were also found to be significant (db-RDA *p* value < 0.01). On the other hand, db-DRA was able to single out a small set of condition-specific phylotypes (Additional file 4: Figure S2b). A few select examples are shown in Fig. 3b. Unclassified phylotypes in the *rc4–4* genus (*Peptococcaceae* family) and the *RF39* order were indicative of non-irradiated states, with variations in relative abundance that never regained control levels for the samples profiled in this work. Relative abundance variations at 0.1 Gy reached a maximum at 10 days for *A. muciniphila*, while at 30 days, these low-dose samples are better characterized by the overabundance of the *Clostridiales* order (*unclassified* and *Lachnospiraceae* species along with *Ruminococcus gnavus*, among others).

Overall, model-based classification and db-RDA at the phylotype level suggest a model in which different radiation doses initiate a distinct reorganization of the microbial composition. In fact, phylotypes with significant association with a given condition showed good performance as condition-specific classifiers in receiver operating characteristic (ROC) analysis (Fig. 3c). This new dose-dependent state seems to be followed by a time-modulated transition towards a new, yet unknown ecological equilibrium post-irradiation.

### Microbial alterations contribute to functional shifts after irradiation in mice

**Fig. 4:**
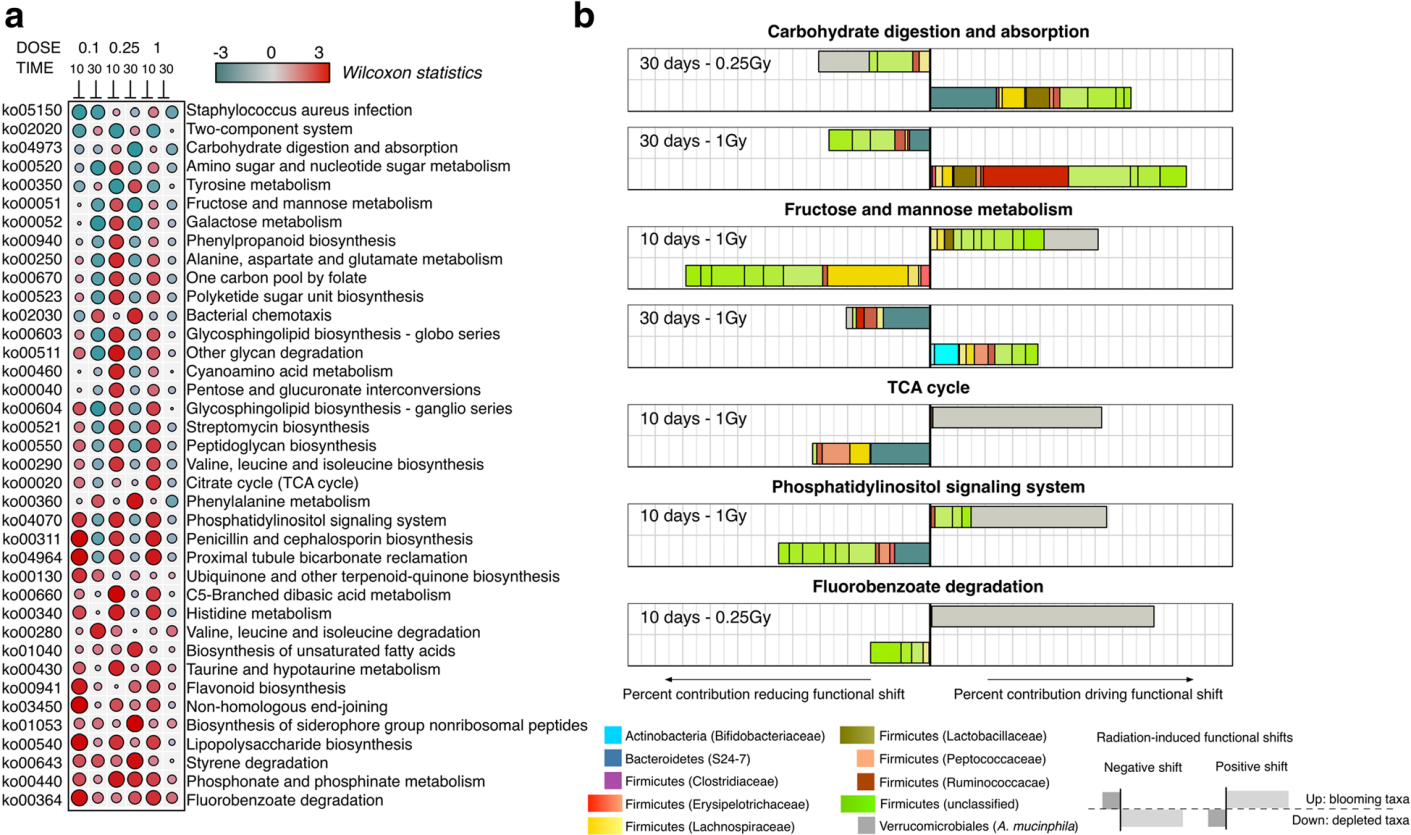
Functional shifts within the irradiated microbiome. **a** Summary of significant functional shifts predicted by the *FishTaco* approach. For each KEGG pathway and each dose, shown is the magnitude *W* (Wilcoxon test statistics, highlighted by *color* and proportional to circle size) of the predicted functional shift with respect to time-matched, non-radiated controls. Net positive shifts (*red*) refer to higher pathway activity in irradiated samples. Net negative shifts (*green*) are the result of lower pathway activity in irradiated samples. **b** Deconvolution of significant community-wide functional shifts into individual taxonomic contributions. Only explicit contributions (taxa with enzymatic activity in the pathway) are shown for greater clarity. For each example, the *top barplot* represents relative contributions to net functional shifts in (**a**) for all to taxa with higher abundance in irradiated samples (resp. lower for bottom barplot)

Our next goal was to determine if the observed variations in radiation-responsive taxa contribute to community-wide functional shifts. In order to account for the compositional nature of the data, we employed the *FishTaco* framework [15], a recently developed approach that deconvolves predicted functional shifts into taxon-level contributions along with their statistical significance (see Methods for details). Figure 4a shows the net magnitude *W* (Wilcoxon test statistic) for predicted shifts in irradiated samples as compared to non-irradiated, time-matched samples (full results are provided in Additional file 7: Table S6). These results predict that the functional potential of the gut microbiome is pushed far from its equilibrium even at low doses of high LET radiation and that this departure from the equilibrium seems to mimic the dose-dependent behavior observed at the species level.

Predicted functional shifts were further examined for their association with the relative extinction or blooming of specific phylotypes (Fig. 4b). We observed a marked downregulation of *carbohydrate digestion and absorption*, a pathway in the mammalian gut that is largely dependent on microbiome-specific reactions. Here, this drop in functional capacity 30 days post-irradiation could be tracked down to the depletion of some *Bacteroidetes* and *Lactobaciliaceae* phylotypes for 0.25 Gy, while for higher doses, the major contribution to this shift can be attributed to the depletion of *Erysipelotrichaceae* phylotypes. In both cases, the increased abundance of phylotypes with enzymatic potential in this pathway (e.g., *Verrucomicrobiales* at 0.25 Gy, *Ruminococcaceae*) is not able to fully compensate the predicted reduced activity in this pathway (Fig. 4a). An example of a ubiquitous pathway reflecting the strong fluctuating abundance of several taxa is *Fructose and mannose metabolism* (Fig. 4a, b). The early response (10 days) for the enzymatic abundance in this pathway (upregulation) can be attributed in part to *Verrucomicrobiales* and *Lactobaciliaceae* phylotypes, due to their elevated abundance relative to other prevalent *Firmicutes* taxa. However, we found a consistent drop in activity 30 days after irradiation, which for high doses is significantly associated to the extinction of *Bifidobacteriaceae* phylotypes. Another striking example are the variations observed for the activity within the tricarboxylic acid (TCA) cycle, which seemed to result from the overabundance of *A. muciniphila* (*Verrucomicrobiales*) phylotypes that prevailed over the depletion of highly abundant, otherwise inhabitants of the normal microbiota (*Bacteroidetes*, *Lachnospiraceae*, *Peptococcaceae*, and *Rumincoccaceae* among others, Fig. 4b).

The previous activity pattern (early upregulation followed by a decrease pathway activity at 30 days) was observed in numerous cases (Fig. 4a) and is likely to be a consequence of a transient dysbiotic microbiome after radiation exposure. However, the long-term clinical consequences of such functional shifts in the host are largely unknown and will probably be a function of the duration of this transient state and the stability of the altered microbiota.

### Perturbations in the metabolome of mice exposed to low-dose high LET radiation

Next, we hypothesized that irradiated samples could be characterized by the differential abundance of specific metabolic products and that some of the metabolic perturbations would correlate with the changes observed in the gut microbiome. Hence, we interrogated the fecal metabolome from the same mice using untargeted metabolic profiling. We detected more than 4500 features by LC-MS and compiled putative annotations based on accurate mass from various databases (see Methods and Additional file 8: Table S7). We first aimed to produce an unsupervised classification of metabolite abundance profiles, in order to look for potential parallelisms with the previously described variations in the microbiome. Multivariate regression followed by unsupervised clustering confirmed that highly variable features (FDR < 10e^−4^ for at least one covariate in the linear regression model) showed similar dose-dependent responses (Additional file 4: Figure S3a). In particular, a significant fraction of highly variable features (284 out of 331) were regulated for the lowest dose (0.1 Gy), and 152 features were statistically significant at this dose only.

The identities of significantly dysregulated metabolites were confirmed using tandem mass spectrometry (see Methods). We again observed several classes of features with moderate or no response for the highest dose employed (e.g., cluster 7 in Additional file 4: Figure S3a). Metabolites classified in the latter cluster were preferentially annotated in central metabolic pathways (*Glycolysis and gluconeogenesis*, *Fructose and mannose metabolism*, *Pyrimidine metabolism*, *Lineloic acid metabolism*, Additional file 8: Table S7). Therefore, the metabolic turnover of the gut ecosystem seems to be significantly altered at low radiation doses.

**Fig. 5:**
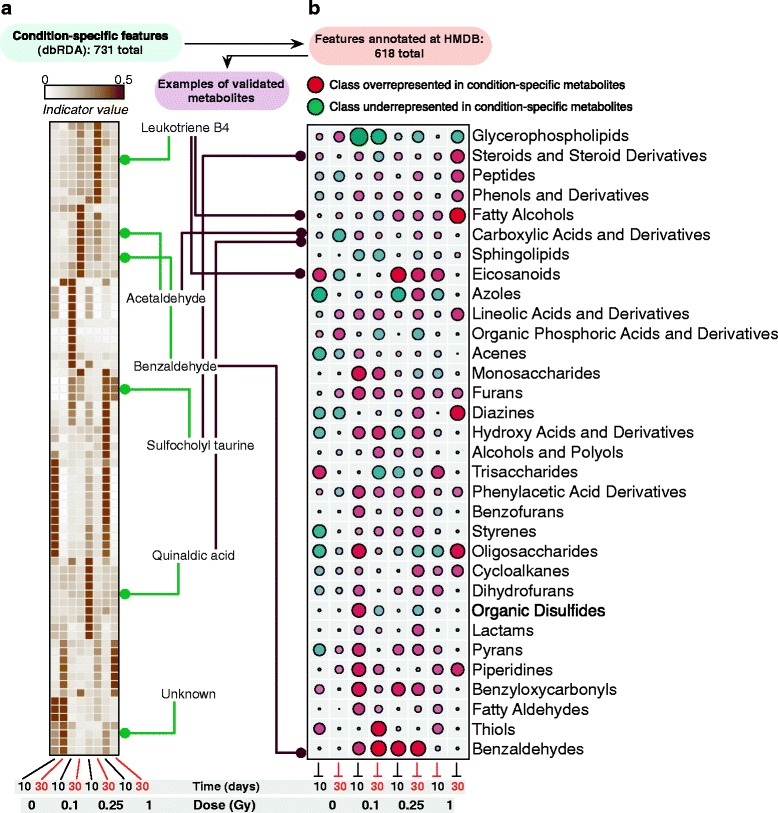
Metabolic classifiers and shifts within the irradiated metabolome. **a** Heatmap of per-group indicator values (distance-based redundancy analysis; db-RDA) for selected condition-specific features. The total number of condition-specific features (out of a total of ~ 4500) is highlighted. **b** Enrichment analysis of condition-specific putatively annotated metabolites in metabolite classes from the HMDB chemical taxonomy database. Over-represented classes (*red*) are those with higher relative presence in the set of condition-specific metabolites as compared to the entire metabolomics dataset (respectively lower for under-represented classes in *green*). *Circle size* is proportional to the (unsigned) fold ratio between those relative abundances

Additionally, db-RDA analysis was able to isolate condition-specific features, which in their turn provided a more discrete account of metabolic shifts across our dataset (Fig. 5). We compiled *chemical taxonomy information* (HMDB database) for the pool of features with putative annotations and summarized enrichment results at the *class* level for condition-specific molecules (Fig. 5, right panel; Additional file 4: Figure S3b, and Additional file 9: Table S8). Among prevailing metabolite classes, precursors of glycerophospholipids, typically regarded as a fingerprint of healthy gut metabolism [16], were found to be under-represented among the classifiers of radiated samples. Besides, a number of metabolite classes were over-represented in classifiers of irradiated samples (*Aldehydes*, *Derivatives of Phenylacetic acid*, and *Eicosanoids*, among others). Specifically, for intermediate doses of ionizing radiation (0.1 and 0.25 Gy), spectral features annotated as leukotriene B4, acetaldehyde and benzaldehyde, or auinaldic acid were among the most significant classifiers. On the other hand, sulfocholyl taurine showed high indicator value for 1 Gy samples, concurrent with an observed shift towards steroids and derivatives for the same samples.

Therefore, untargeted metabolomics lend credence to the widespread metabolic shift predicted from variations in microbial species, which in turn has an impact in a heterogeneous array of gut signaling pathways. Although a substantial number of spectral features could not be annotated in current metabolomics reference databases, these results reveal a radiation-induced breakdown in the symbiotic homeostatic control of several gut metabolic pathways and provide insights for future mechanistic and interventional studies.

### Metabolic network modeling and microbiome-metabolite associations

The impact of the microbiome on the host’s metabolic activity has been extensively studied and reviewed, and is known to affect both local and systemic metabolism [17, 18]. Moreover, the individual or collective contribution of microbial taxa to specific metabolic pathways has been established in numerous settings [17, 19]. However, the functional redundancy of the gut microbiome and the complex interactions along the host-microbiome axis can result in a separation between microbial composition and overall metabolic turnover [20, 21]. We have shown above that high LET radiation induces (1) significant changes in the fecal microbial composition, concomitant with a shift in its predicted functional potential and (2) shifts in the combined host-microbiome metabolic output. We next aimed to integrate our data to establish whether radiation-induced alterations in microbial composition (community structure) can predict variations in specific metabolic shifts (community metabolism). To this end, we employed metabolic network modeling [22–24] to estimate the community-wide metabolic output of our inferred metagenomes and compared these predictions with the abundance of metabolites (validated using tandem MS), in our LC-MS dataset.

**Fig. 6:**
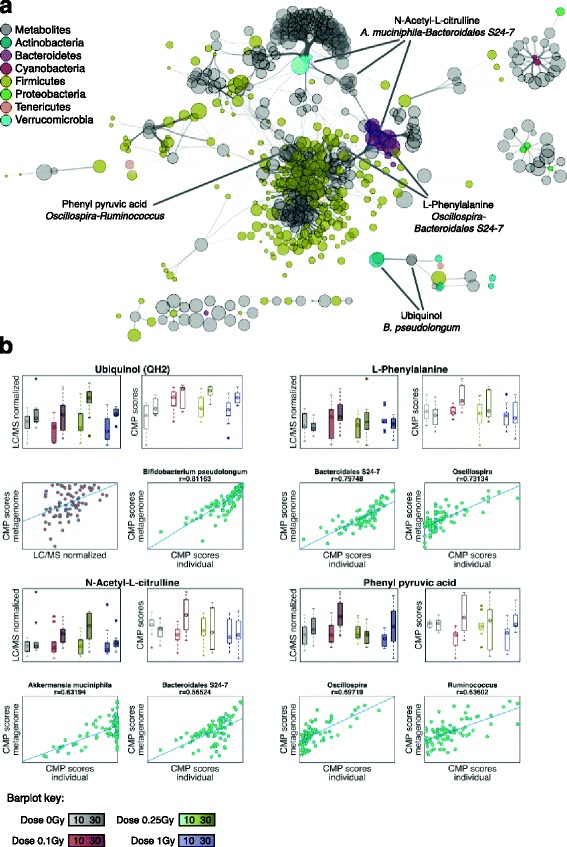
Metabolic network modeling and taxa-metabolite associations. Multi-omics (16S and LC-MS) data integration was performed under the Predicted Relative Metabolic Turnover (PRMT) framework. **a** Network visualization of significant associations between well-predicted metabolites (Mantel *p* value < 0.01 and FDR < 0.01, a total of 259 compounds) and bacterial phylotypes with a significant contribution to community-wide CMP scores (correlation between individual and community-wide CMP scores > 0.5 for a given metabolite, a total of 265 phylotypes). Node size is proportional to the relative abundance of the corresponding metabolite (from LC-MS) or phylotype (from 16S amplicon data). Edge width is proportional to the strength of association between each metabolite-phylotype pair (as measured by the correlation above). Highlighted are examples of well-predicted metabolites with significant agreement between experimental and predicted relative abundances and their association with specific phylotypes. **b** For each well-predicted metabolite highlighted in (**a**): *solid barplots* represent actual relative abundances (LC-MS); *hollow barplots* represent “predicted” relative abundances (CMP scores); *red scatterplot* for ubiquinol shows the correlation between actual and predicted relative abundances across all samples; *green scatterplots* show the correlation between community-wide and individual taxa contributions to predicted relative abundances, for taxa classified as key drivers of variations in metabolite relative abundances

We mapped our inferred metagenomes and metabolite-putative annotations to a reference set of enzymatic reactions retrieved from the KEGG database [25, 26] and implemented a modeling framework based on *Community-Based Metabolite Potential* (CMP) scores [24]. These scores were used as a surrogate for the relative capacity of the inferred metagenome to produce or deplete the metabolite and enabled us to identify a set of *well-predicted metabolites* by direct comparison to actual metabolomics data (see Methods). Strikingly, we found that ~ 30% of the mapped compounds were classified as well predicted (Mantel *p* value and FDR *q* value < 0.01). Although this degree of predictability compares with previous results in both vaginal and fecal samples [24], pairwise correlations between CMP scores and LC-MS abundances across all samples were lower than previously observed, which could be a consequence of the complex host-microbiome post-radiation dynamics described above. In fact, metabolites categorized as well predicted (see examples in Fig. 6) showed high concordance between actual and predicted metabolite abundances, particularly for samples with outlier values, and even for moderate values of the global correlation across all samples. This underscores the connection between strong variations in microbial abundances and metabolic output in our system.

Among the set of well-predicted metabolites, we observed enrichment in metabolite classes strongly associated with bacterial activity in the gut (*amino acids and derivatives*, *steroids and steroid derivatives*, *prenol lipids* and *carboxylic acids and derivatives*, along with *carbohydrates and carbohydrate conjugates*; Additional file 10: Table S9). Overall, these results indicate that predicted metagenome profiles, post high LET radiation exposure, can be used to explain a significant fraction of the observed variance in metabolic output.

To further explore the association between bacterial species and metabolites, we identified the phylotypes with significant individual contributions to the community-wide CMP scores (see Methods and Additional file 10: Table S9). Figure 6a shows the network of associations between well-predicted metabolites and significant contributors to the predicted community-wide CMP scores. Interestingly, the structure of this network mainly reflected the functional specificity of phylotypes in different bacterial families, as they were primarily associated with distinct sets of well-predicted metabolites. As expected, the diverse *Firmicutes* family contributed a significant and redundant array of associations, with some anticipated overlap with *Bacteroidetes* phylotypes. Also, and in concordance with the functional compensatory effects observed from our predicted metagenomes between *Verrucomicrobia* and *Bacteroidetes* phylotypes (Fig. 4), these two families were predictive of variations in a common set of metabolites. For instance, citrulline abundance in fecal samples has been shown to correlate with the abundance of *Bacteroides* and other gut bacterial species [16, 27]. Here, we found that N-acetyl-L-citrulline CMP scores correlated with *Bacteroidales spp.*, while *A. muciniphila* was particularly associated with the lowest CMP scores (Fig. 6b). Among the set of well-predicted metabolites with the highest global correlation, we observed an increase in the abundance of ubiquinol particularly for samples 30 days after exposure. The individual CMP scores for several *Actinobacteria* species were strongly correlated with community-wide CMP scores for ubiquinol, with *Bifidobacterium pseudolongum* being classified as the top key contributor. Additional examples of well-predicted metabolites include *Phenylalanine*, which was found to be significantly associated with *Bacteroidales* species, although *Oscillospira* was particularly predictive for samples with the highest levels of Phenylalanine (Fig. 6b)*.* Finally, phenylpyruvic acid is a bacterial byproduct of phenylalanine metabolism. Our data suggest a preeminent role of Ruminococcaceae species (*Oscillospira* and *Ruminococcus* among others) in high levels of this metabolite. Finally, we found that a number of well-predicted metabolites in this study (phenylalanine, citrulline, phenylpyruvate, chenodeoxycholate, and mannose among others) were also well predicted in metabolic models of inflammatory disorders [24].

In summary, the enzymatic potential of the irradiated microbiome is a good predictor of the metabolic output. Combined, the observed functional and metabolic shifts parallel previous observations on the relationship between dysbiosis of the gut microbiota and disease, as discussed below.

## Discussion

In this study, we report the results from a murine model-based study aimed at delineating the modulation of the gut microbiome and metabolome after exposure to different levels of ionizing radiation. Somewhat unexpectedly, our model showed a higher sensitivity of the gut ecosystem to lower doses—0.1 and 0.25 Gy as compared to the highest dose—1 Gy. This threshold-like response was recapitulated at the (predicted) functional and metabolome levels. Although the basis for this behavior in our system is uncertain, non-linear responses in the range 0.1–1 Gy have been previously reported and explained in terms of a transition from low-dose hyper-radiosensitivity (HRS) to increased radioresistance (IRR) at doses of ~ 0.3 Gy in mammalian cells (initially observed by Marples et al. [28] and extensively reviewed thereafter [29]). The HRS regime seemed to be indicative of adaptive-like responses aimed to block low-dose damage propagation from DNA to cells and tissues. These could involve time-delayed signals that can span from hours (e.g., scavenging of toxins) to months (e.g., immune responses). At higher doses, DNA repair mechanisms were fully in effect and resulted in a seemingly reduction in radiosensitivity, or IRR. Here, we have presented data in the range 0–1 Gy and up to 30 days after radiation; our results suggest an emergent HRS/IRR behavior in the complex gut ecosystem, where protective signals in the range 0.1–0.25 Gy would be able to induce ecological, functional, and metabolic shifts in the gut that are not present under an IRR regime at 1 Gy. The behavior of the gut ecosystem under substantially higher doses than those employed here (such as 5 Gy and above) remains an open question.

The basis for the observed convoluted response to radiation remains elusive. For high LET radiation, one could speculate on the presence of modulatory factors at high doses. These would include a protective DNA repair and oxidative phosphorylation signaling pathway response of the microbial ecosystem [30, 31] amplified by the local hematopoietic or epithelial cellular response [32]. On the other hand, host-specific alterations to this dose and type of radiation are poorly understood, although recent gene expression studies indicate that there is a detectable and consistent protective-like response [30]. A core of signaling radiation-responsive pathways includes those involved in sensing alterations in redox balance and downstream regulatory activities to restore homeostasis, e.g., cell-cycle modifications, cytotoxicity, and inflammatory responses [31]. Our data shows a long-term modulation of the gut ecosystem; at least 30 days after a single (high dose rate) exposure to radiation. Therefore, it is tempting to speculate that radiation-induced redox imbalance is followed by a pro-inflammatory dysbiotic state, as a mechanism with the ability to sustain a modulatory effect on that time scale. We profiled plasma samples from C57Bl/6 J male mice that were exposed to ^16^O (600 Mev/n) at 14 and 90 days after irradiation. We found an increase in plasma levels of phosphatidic acid (PA) and lyso PA metabolites that are known to activate pro-inflammatory mTOR signaling directly (data not shown) [33]. On the other hand, untargeted metabolomics data suggest that the metabolic turnover of the gut ecosystem was substantially altered for low radiation doses, including a number of spectral features with dose-dependent abundance and potential host metabolic impact: leukotriene B4 [34], phenyl acetic acid [35–37], sulfocholyl taurine [38], and the L-tryptophan metabolites quinaldic acid and kynurenic acid [39, 40]. Regardless, these observations only provide indirect evidence of host-microbiome interactions, and therefore, additional studies will be required to fully characterize the crosstalk between host-derived signals and the observed dose-dependent responses to radiation reported here.

Alternately, we employed metabolic network modeling to delineate the association between gut microbial ecology and the collective, host-microbiome metabolic output after irradiation. In agreement with previous studies, we found that the abundance of a non-marginal fraction of metabolites can be predicted by microbial community structure. Examples of well-predicted metabolites include ubiquinol, whose abundance can be modulated as a response to accumulated oxidative stress or DNA damage [41]. In agreement with our modeling results, the overabundance on Phenylalanine has been previously associated with the activity of *Clostridiales* species [42]. Another example of well-predicted metabolite was N-acetyl-L-citrulline, which has been mechanistically linked to radiation-induced gut epithelial loss [32]. Finally, phenylpyruvic acid is a bacterial byproduct of phenylalanine metabolism and could be a fingerprint of microbial-enhanced fermentation [43]. Therefore, our integrative approach supports the notion that microbiome-mediated changes in the metabolite milieu could play a key role on host-microbiome interactions post-radiation.

Functional resilience is an important quality of the microbial ecosystem [44] and can be altered by metabolic cues [45–47], bacterial phage activation [48, 49], and other heterologous competitive relationships [50–52]. Our analysis of predicted functional shifts allowed us to evaluate the impact of specific microbiome compositional variations. Of note, several pathways dominated by microbiome-specific enzymatic reactions (*Lipopolysaccharide Biosynthesis*, *Fluorobenzoate Degradation*, *Phosphonate and Phosphinate Metabolism*, *Taurine and Hypotaurine Metabolism*) were predicted to be constitutively upregulated in irradiated samples. In other cases, predicted functional variations were reflective of the opportunistic behavior of several taxa like *A. muciniphila*, *Ruminococcus gnavus*, and *Erysipelotrichaceae*, among others. Their pronounced overabundance even at low doses paralleled a transient abundance decline of commensals (such as *Actinobacteria*, *Bacteroidetes*, *and Firmicutes*). Both *A. muciniphila* and *Erysipelotrichaceae* have been deemed as opportunistic gut colonizers after antibiotic treatment [53–55]. Also, *A. muciniphila* has gained much recent attention because of its overabundance in response to various environmental triggers [55–62]. In particular, *A. muciniphila* has been regarded as a colitogenic and pro-inflammatory species in specific models of colitis [63, 64] through its ability to degrade the intestinal mucus layer and high immunostimulatory activity. However, within the complex gut ecosystem, those findings are likely to be context dependent [65, 66]. Our study does not rule out the possibility that these and other observed changes are the result of mutualistic protective responses to harmful alterations Therefore, the long-term consequences of a transient colonization by *A. muciniphila* and other species after a short-term insult remain uncertain.

The previous remarks raise a number of open-ended relevant questions, in particular about the transient or permanent nature of a radiation-induced dysbiotic state under space travel conditions. First, and due to experimental constraints, the ^16^O exposures in this study were performed at high dose rates, since chronic or fractionated exposures to oxygen ion irradiation were not feasible at the time of these studies. However, heavy ion radiation in space occurs continuously and at lower dose rates, and although a reduced diversity on the microbiota has been reported following space flight [67], little is known about how the microbiome and its metabolic output are modulated under chronic, low dose rate exposures. Additionally, space travel conditions constitute a highly unusual environment for the microbiome. The lack of exposure to microbial diversity due to a limited diet and extremely sterile habitat could amplify the effect of harmful, opportunistic pathogens [68], or impede the correction of an otherwise transient dysbiotic state. Studies are under way to evaluate the feasibility of dietary interventions to improve astronaut health [68]. However, the translational potential of our findings regarding exposure to high LET radiation should be studied in the future. Of particular relevance for space travel applications, it would be informative to extend the scope of our integrative approach by evaluating additional tissue function outcomes from the same cohort, such as intestinal structure, cognitive function, and cardiovascular function and structure. Still, our data show for the first time that even small doses of high LET radiation constitute a challenge to the functional resilience of the gut ecosystem.

## Conclusions

Our integrative analysis underscored several points; firstly, there were robust changes in ecological communities harboring the gut microbiota as a consequence of high LET exposures (16 O); secondly, these changes seem to shift the equilibrium towards an increase in opportunistic pathogens with a concomitant decrease in normal microbiota upon irradiation; finally, these changes were predicted to induce functional shifts in metabolism, both at the level of the predicted enzymatic potential of the perturbed microbiome and of the metabolome. Most importantly, metabolic network modeling showed that specific changes in the metabolome are connected to irradiation-induced changes in the abundance of specific taxa. Our model suggests an emergent, dose-dependent hyper-radiosensitivity behavior of the gut ecosystem. Regardless of the specific mechanisms involved in these singular responses, our exploratory study clearly establishes that high LET radiation induces a metabolite-mediated, convoluted reorganization of the gut ecosystem. Therefore, the implication of microbiome-mediated host pathophysiology after low-dose ionizing radiation may be an unappreciated biologic hazard of space travel and deserves experimental validation. This study provides a conceptual and analytical framework to increase our understanding of the chronic effects of space radiation on human health.

## Methods

### Animal and irradiation protocols

Male C57BL/6J mice (Jackson Laboratory) were purchased at 4 weeks of age and housed at the Division of Laboratory Animal Medicine, University of Arkansas for Medical Sciences (UAMS), on a 12:12 light-to-dark cycle with free access to food (soy-free rodent diet 2020X, Harlan Teklad) and water. At 6 months of age, mice were transported to Brookhaven National Laboratories (BNL) and housed under comparable conditions (12:12 light-to-dark cycle, free access to rodent diet 2020X and water). After a one-week acclimation period, mice were individually placed in well-ventilated clear Lucite cubes (3 × 1½ × 1½ in.) and exposed to whole-body ^16^O irradiation (600 MeV/n; 0.1, 0.25, or 1.0 Gy, 0.21–0.28 Gy/min) at the NASA Space Radiation Laboratory. Sham-irradiated mice were placed in the same holders, but were not exposed to radiation. A total of 10 mice per dose group were used. Dosimetry details and schematics along with dose distribution curves are reported elsewhere [69]. One day after (sham-) irradiation, all mice were returned to UAMS and placed on 2020X diet containing 0.68 g/kg fenbendazole (Harlan Teklad) as part of the standard UAMS rodent quarantine procedure. At 10 and 30 days after irradiation, mice were individually placed in a Plexiglas box to obtain fresh fecal pellets. Fecal pellets were stored at −80 °C until processing. Each pellet was divided into two parts under liquid nitrogen, one halve was shipped to the University of California Los Angeles for 16S rRNA amplicon sequencing and the other halve to Georgetown University for metabolomics.

### 16S rRNA amplicon sequencing library preparation

Genomic DNA was extracted using the PowerSoil DNA Isolation Kit (MO BIO Laboratories, Carlsbad, CA, USA) with a 30-s beat-beating step using a Mini-Beadbeater-16 (BioSpec Products, Bartlesville, OK, USA). Polymerase chain reaction amplification of bacterial 16S rRNA genes was performed using extracted genomic DNA as the template. The 100 μl reactions contained 50 mM Tris (pH 8.3), 500 μg/ml bovine serum albumin, 2.5 mM MgCl_2_, 250 μM of each deoxynucleotide triphosphate, 400 nM of each primer, 4 μl of DNA template, and 2.5 units JumpStart Taq DNA polymerase (Sigma-Aldrich, St. Louis, MO, USA). The PCR primers (F515/R806) targeted the V4 hypervariable region of the 16S rRNA gene, with the reverse primers including a 12-bp Golay barcode. Thermal cycling were performed in an MJ Research PTC-200 (Bio-Rad Inc., Hercules, CA, USA) with the following parameters: 94 °C for 5 min; 35 cycles of 94 °C for 20 s, 50 °C for 20 s, and 72 °C for 30 s; 72 °C for 5 min. PCR products were purified using the MinElute 96 UF PCR Purification Kit (Qiagen, Valencia, CA, USA). DNA sequencing was performed using an Illumina HiSeq 2500 (Illumina, Inc., San Diego, CA, USA), in paired-ended mode. Clusters were created using template concentrations of 4 pM and PhiX at 65 K/mm^2^. Sequencing primers targeted 101 base pair reads of the 5′ end of the amplicons and 7 base pair barcode reads. Reads were filtered using the following parameters: minimum Q-score—30, maximum number of consecutive low-quality base calls allowed before truncating—3, and maximum number of N characters allowed—0. All filtered V4 reads had a length of 150 bp.

### Analysis of 16S rRNA amplicon sequencing data

De-multiplexing and paired-end joining of 80 sequencing libraries was performed in QIIME [70] using default parameters. Sequencing reads were classified and summarized at different phylogenetic levels down to Operational Taxonomic Units (OTUs) [71, 72] using a similarity threshold of 97% within the GreenGenes [73] v13_8 reference database. One sample with less than 60,000 classified sequences was removed. The average number of OTUs detected per sample was 862.4 ± 88.4, and the mean counts per sample was 100,745.5. The number of detected OTUs (counts > 0 in at least one sample) was 7377, for an OTU table density of 0.117. Therefore, independent filtering was applied as recommended for Illumina amplicon data [74] by removing low abundance OTUs (those with < 0.0005% of reads in the total dataset). The resulting matrix provides a highly replicated, deeply sequenced dataset with 1260 OTUs (average number of OTUs detected per sample 718.1 ± 60.0, mean counts per sample = 100,536.4, final OTU table density of 0.57), which allowed us to perform differential abundance analysis with increased detection sensitivity. Downstream analysis (see below) was always performed from randomly rarefied tables at a depth of 60,000 reads per sample.

In light of the distinct effect that different doses had on microbial diversity, we were primarily interested on modeling ordered, monotonic changes to radiation. Downstream analysis of 16S rRNA amplicon data was therefore always carried out in terms of categorical variables for Time and Dose. QIIME [70] was employed for the ecological analysis of 16S rRNA data, including relative abundance of taxa, and alpha and beta diversity analysis. Alpha diversity was estimated using Faith’s phylogenetic diversity metric (PD) as the average across ten different rarefactions of the OTUs count matrix. Differences in diversity levels between groups were tested using a nonparametric two-sample *t* test (999 Monte Carlo permutations). Samples ordination based on beta diversity was examined by means of principal coordinate analyses (PCoA) with phylogeny-based (UniFrac) unweighted distances. Jackknifed analysis on randomly rarefied data along with PERMANOVA and ANOSIM were used to test for significant differences in beta diversity between factors of the experimental design. Similarly, the Kruskal-Wallis test was used to evaluate the effect of the experimental factors on the relative abundance at different taxonomic levels. Additional ordination and discriminant analysis was performed by means of *distance-based redundancy analysis* (*db-RDA*) using the *vegan* [75] package in R. Negative binomial statistics were employed to identify differentially abundant taxa and classify them in groups with similar abundance profile, with increased detection sensitivity for rare taxa. In particular, *DESeq2* [76] was first used to fit the count data to different models: an *additive model* (*~ Time + Dose*), two *reduced models* (*~ Time* or *~ Dose*) and a full *interacting model* (*~ Time + Dose + Time:Dose*). The results from these models were compared for each taxa using ANODEV to capture statistically significant responses to experimental factor, their combination and/or their interaction. All taxa that tested significant (adjusted *p*-value < 0.05) in at least one contrast were pooled. This target pool was then subjected to model-based clustering using *MBCluster.Seq* [77] to classify taxa based on their overall abundance profile.

*PICRUSt* [78] was used to predict the metagenome in terms of Kegg Orthology (KO) terms for each 16S rRNA sample. The output from PICRUSt was further normalized using *MUSICC* [79] for downstream analyses, obtaining both intra- and inter-sample corrections. Microbiome functional shifts and phylotype-level contributions to functional shifts were obtained using the *FishTaco* framework [15]. Input for FishTaco included a pre-computed OTU-KO table from the PICRUSt analysis, output from MUSICC, and OTU relative abundances. FishTaco was run on multi-taxa mode for each pairwise comparison between irradiated and non-irradiated samples. For each KEGG pathway, we estimated both positive and negative functional shifts using two different metrics (Wilcoxon and log-ratio tests). In order to overcome the computational cost of the FishTaco deconvolution approach, we estimated the functional shifts for the top 100 phylotypes with the maximum relative abundance across our dataset, and the set of all possible independent tests were analyzed in parallel in a computer cluster. FishTaco deconvolves each functional shift in pairwise case vs. control comparisons into four different modes: (1) case-associated taxa driving functional shift (taxa over-represented in cases with enzymatic activity in pathway); (2) case-associated taxa reducing functional shift (taxa over-represented in cases but with no enzymatic activity in pathway); (3) control-associated taxa driving functional shift (taxa over-represented in controls with no enzymatic activity in pathway); and (4) control-associated taxa reducing functional shift (taxa over-represented in controls with enzymatic activity in pathway). Figure 4a shows the net functional shift in terms of Wilcoxon test statistics. For greater clarity, Fig. 4b summarizes taxon-level percent contributions to the net functional shifts only for phylotypes with functional activity in the pathway.

### Fecal metabolomics using UPLC-ESI-QTOF-MS

Fecal samples were processed by initially homogenizing in extraction solvent containing 50% methanol, 30% isopropanol, and 20% chloroform and internal standards [80]. The samples were centrifuged and chilled 1:1 acetonitrile was added to the Eppendorf vials. The samples were incubated at −20 °C overnight to allow protein precipitation followed by centrifugation. The supernatant was combined and dried under vacuum and resuspended in water containing 50% methanol for MS analysis. The sample queue was randomized to avoid bias. Each sample (2 μl) was injected onto a reverse-phase 50 × 2.1 mm Acquity 1.7 μm BEH C18 column (Waters Corp, Milford, MA) using an Acquity UPLC (Waters Corporation, USA) system online with an electrospray quadrupole time-of-flight tandem mass spectrometer (ESI-Q-TOF) (Xevo–G2, Waters Corporation USA) operating in positive and negative ion mode, the details of tune page parameters have been described before [81–83]. A 0.2 ng/ul/μL solution of Leucine-Enkaphlin in 50% acetonitrile in water ([M + H] ^+^, m/z 556.2771 and [M-H]^−^, m/z 554.2615) was infused at 5 μL/min flow rate as the reference mass (lock mass) for accurate mass measurements. The quality control (QC) samples for each matrix comprised an aliquot of all samples in the study set, thus representing a universal set of metabolites. Initially the column was conditioned using this QC sample and thereafter it was injected after every ten injections to account for reproducibility of the LC-MS data [84]. The overlay of total ion chromatograms showing chromatographic reproducibility and mass error using mixture of standards (metmix) is detailed in Additional file 4: Figure S4.

All initial analyses were performed with putative annotated metabolites; however, a subset of significantly dysregulated metabolites was subsequently confirmed by tandem mass spectrometry (see Computational analysis of metabolomics data). The UPLC-QTOF raw data files were converted into NetCDF format (Network Common Data Form) using the data bridge function incorporated in the MassLynx software (Waters Corp, Milford, MA). Subsequently, the LC-MS data were preprocessed using XCMS software, as has been described [85]. R script used for data pre-processing is provided in Additional file 4. The data were normalized to the ion intensity of the internal standards (debrisoquine and 4, Nitrobenzoic acid) and weight of the fecal pellet.

### Computational analysis of metabolomics data

Normalized LC-MS data were employed for all downstream analyses. We employed mass search to assign putative metabolite identifications from the Metlin and HMDB databases [86, 87]. We performed searches for both positive and negative modes with mass tolerance thresholds in the range 1 to 7.5 ppm. The final identification was based on either minimal mass difference or manual curation using the fragmentation spectrum of the standard metabolites. The identities of all significantly dysregulated metabolites were confirmed using tandem mass spectrometry. The fragmentation information for a subset of metabolites that were significantly dysregulated is included in Additional file 11: Table S10. For metabolic network modeling and metabolite class enrichment, all putative annotations were tested in order to maximize enrichment and overlap with reactions encoded by the inferred metagenome [24], with little differences for different mass tolerance thresholds. Multivariate linear regression, ordination and discriminant analysis were performed as before in R. Enrichment on metabolites classes was performed using chemical taxonomies downloaded from the HMDB database version 3.6.

Metabolic network modeling was performed using the Predicted Relative Metabolic Turnover framework [23] in terms of KEGG enzymatic reactions. We computed Community-wide Metabolic Potential (CMP) scores [24] using in-house scripts in Matlab (R2015a, The MathWorks Inc.). Our implementation was based on a database of irreversible enzymatic reactions from the KEGG database [26] (release 77.1) obtained using the KEGG REST API. The reaction database was represented in terms of a stoichiometric matrix *M*, which links KEGG compound with KO terms. The final normalized form [23] of the matrix *M* (where all positive coefficients are re-scaled to sum 1, or −1 for negative coefficients) represents the relative contribution of each KO gene to the production or depletion of each compound. CMP scores were computed as the matrix multiplication of *M* and *G*, where the latter represents MUSICC-corrected KO-relative abundances. Final integration with metabolomics data was performed by comparing CMP scores to actual LC-MS normalized metabolite abundances, by matching metabolite putative ids with KEGG compound ids. For each metabolite, we performed a Mantel test between the vector of CMP scores and normalized abundance across all samples as before [24]. *P* values from the Mantel test were further corrected for multiple testing using bootstraps to estimate false discovery rates (FDR). Compounds were classified as well predicted if Mantel *p* value < 0.01 and FDR < 0.01. Identification of key phylotypes contributing to a particular CMP score was based on the correlation between community-wide and single-phylotype CMP scores. These were computed as before using a matrix *G* representing the enzymatic content of a single phylotype. Key contributors associated to a given metabolite were selected as the phylotypes with the maximum correlation between community-wide and single-phylotype scores.

## Additional files

Additional file 1: Table 1. Diversity analysis of 16S rRNA sequencing samples. Alpha and beta diversity groups comparison statistics. (XLS 26 kb)

Additional file 2: Table S2. Group significance analysis of 16S rRNA counts at different taxonomic levels. (XLS 61 kb)

Additional file 3: Table S3. Linear discriminant analysis (LDA) effect size (LefSe) analysis of 16S rRNA counts at the phylotype level for the Dose factor. (XLS 57 kb)

Additional file 4: Additional file with supplementary figures. (PDF 14760 kb)

Additional file 5: Table S4. ANODEV and model-based clustering results for 16S rRNA data at the phylotype level. (XLS 836 kb)

Additional file 6: Table S5. Constrained analysis of principal coordinates (db-RDA method) for 16S rRNA counts at the phylotype level. (XLS 671 kb)

Additional file 7: Table S6. Statistical significance of microbiome functional shifts (*FishTaco* algorithm). (XLS 65 kb)

Additional file 8: Table S7. Multivariate regression and clustering of LC-MS data. (XLS 5048 kb)

Additional file 9: Table S8. Constrained analysis of principal coordinates (db-RDA method) for LC/MS data. Enrichment analysis of HMDB metabolite classes. (XLS 5550 kb)

Additional file 10: Table S9. Metabolic network modeling results. (XLS 217 kb)

Additional file 11: Table S10. MS/MS spectral information for significantly altered metabolites irradiated mice. (XLS 19 kb)

## Abbreviations

CAP: Constrained analysis of principal coordinates
CMP: Community-based metabolite potential
db-RDA: Distance-based redundancy analysis
FDR: False discovery rate
FishTaco: Functional shifts taxonomic contributors
GCR: Galactic cosmic rays
GLM: Generalized linear model
Gy: Gray
HMDB: Human metabolome database
HRS: Low-dose hyper-radiosensitivity
IRR: Increased radioresistance
KO: KEGG Orthology
LC-MS: Liquid chromatography-mass spectrometry
LDA: Linear discriminant analysis
LEfSe: Linear discriminant analysis effect size
LET: Linear energy transfer
MS: Mass spectrometry
MUSICC: Metagenomic Universal Single-Copy Correction
OTU: Operational taxonomic unit
PCoA: Principal coordinate analysis
PICRUSt: Phylogenetic Investigation of Communities by Reconstruction of Unobserved States
ROC: Receiver operating characteristic
SPE: Solar particle events
