# Supplementary material for: Space-type radiation induces multimodal responses in the mouse gut microbiome and metabolome
Source: Microbiome. 2017 Aug 18;5:105. doi: 10.1186/s40168-017-0325-z (PMC5563039; doi:10.1186/s40168-017-0325-z)
Supplement: Supplementary file 4 — Additional file with supplementary figures. (PDF 14760 kb) [file 40168_2017_325_MOESM4_ESM.pdf]

Supplementary Information for “Space-type radiation induces multimodal responses in the mouse gut microbiome and metabolome”

A. **List of Supplementary Tables**
B. **Supplementary Figures**
C. **R preprocessing script, XCMS metabolomics tool**

A. List of Supplementary Tables

Supplementary Table 1: Diversity analysis of 16S rRNA sequencing samples. Alpha and beta diversity groups comparison statistics.

Supplementary Table 2: Group significance analysis of 16S rRNA counts at different taxonomic levels.

Supplementary Table 3: Linear Discriminant Analysis (LDA) Effect Size (LefSe) analysis of 16S rRNA counts at the phylotype level for the Dose factor.

Supplementary Table 4: ANODEV and model-based clustering results for 16S rRNA data at the phylotype level.

Supplementary Table 5: Constrained analysis of principal coordinates (db-RDA method) for 16S rRNA counts at the phylotype level.

Supplementary Table 6: Statistical significance of microbiome functional shifts (*FishTaco* algorithm).

Supplementary Table 7: Multivariate regression and clustering of LC-MS data.

Supplementary Table 8: Constrained analysis of principal coordinates (db-RDA method) for LC/MS data. Enrichment analysis of HMDB metabolite classes.

Supplementary Table 9: Metabolic network modeling results.

Supplementary Table 10: MS/MS spectral information for significantly altered metabolites irradiated mice.

B. Supplementary Figures

Figure S1: Pair-wise Linear Discriminant Analysis (LDA) effect size (LefSe). Heatmaps of effect sizes for phylotypes classified as significant by LefSe in at least one pair-wise comparison between irradiated and time-matched, non-irradiated controls. Positive effect sizes correspond to taxa with higher relative abundance in irradiated samples (resp. negative effect sizes for lower relative abundance).

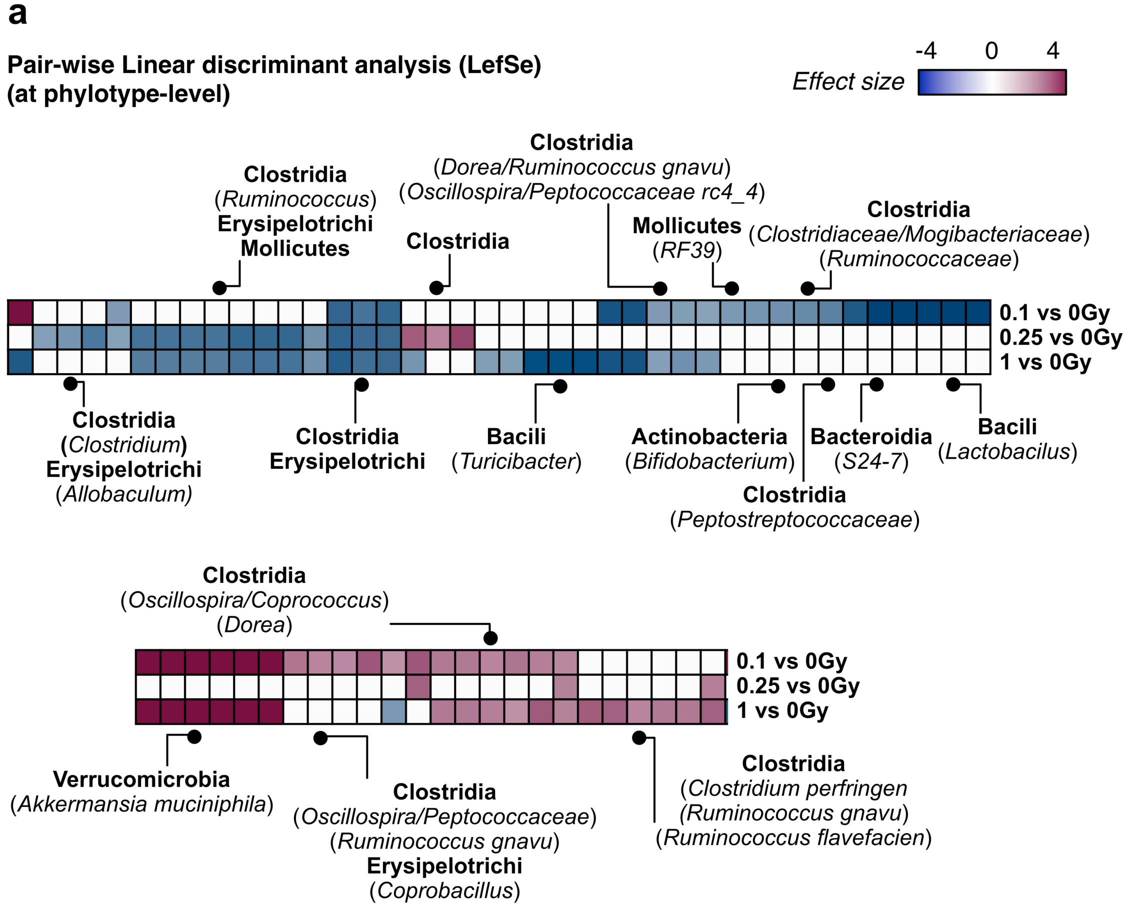

Figure S2: Phylotype-level model-based clustering and db-RDA analysis. (a) Model-based clustering of phylotypes based on overall abundance profiles. Shown are all clusters analyzed in this study along with the number of OTUs classified in each cluster. Line plots represent the average abundance profile for all phylotypes classified in each cluster. **(b)** Heatmap of per-group indicator values (distance-based Redundancy Analysis, db-RDA) for all OTUs profiled in this study.

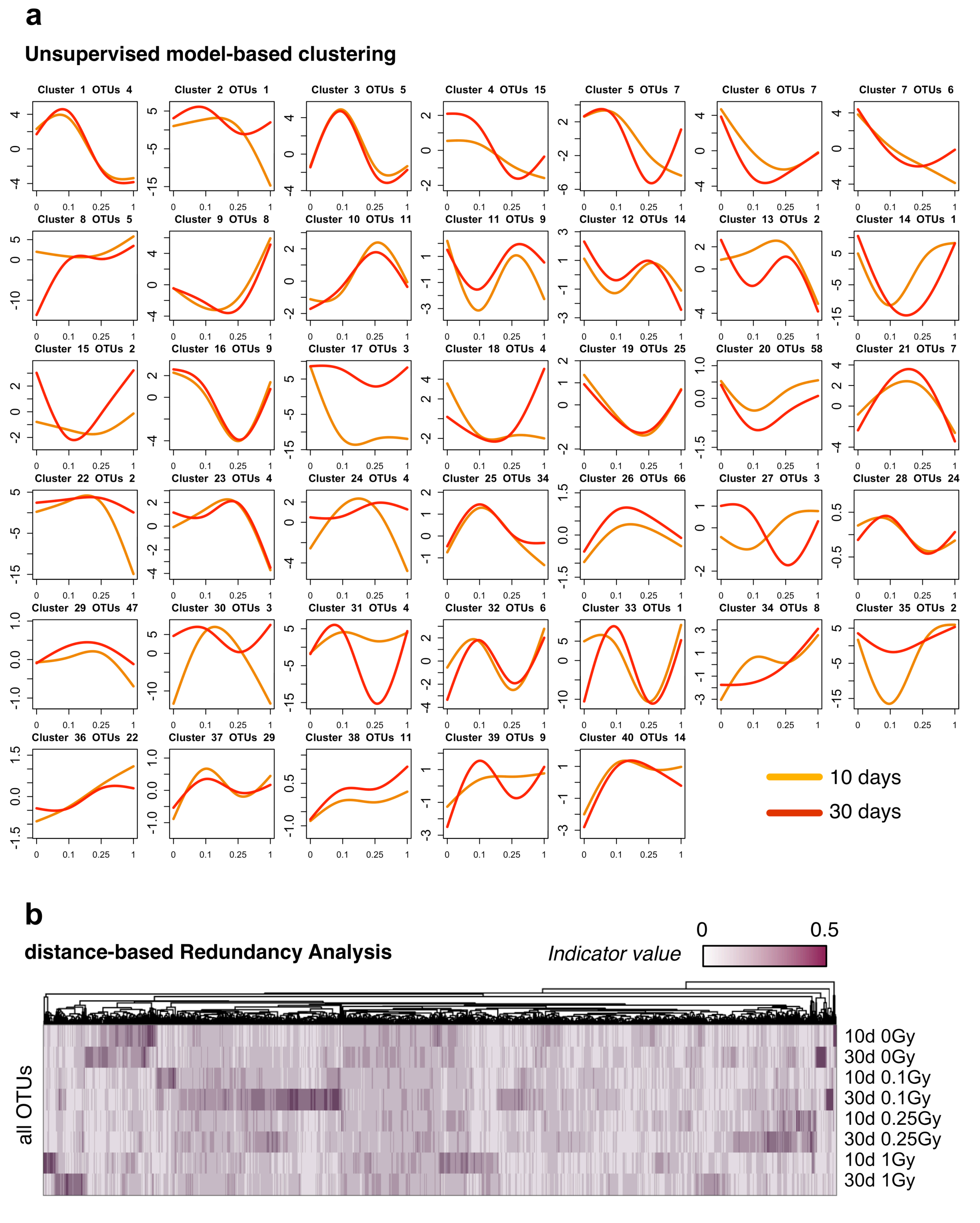

Figure S3: Classification of metabolites according to overall response to irraditation (a) Clustering of metabolites based on overall relative abundance profiles. Only metabolites classified as significant in a multivariate regression analysis were employed. Shown are all clusters analyzed in this study along with the number of metabolites classified in each cluster. Line plots represent the average abundance profile for all metabolites classified in each cluster. **(b)** Barplots of metabolite relative abundances for selected metabolites (Figure 5) with high indicator value (CAP analysis).

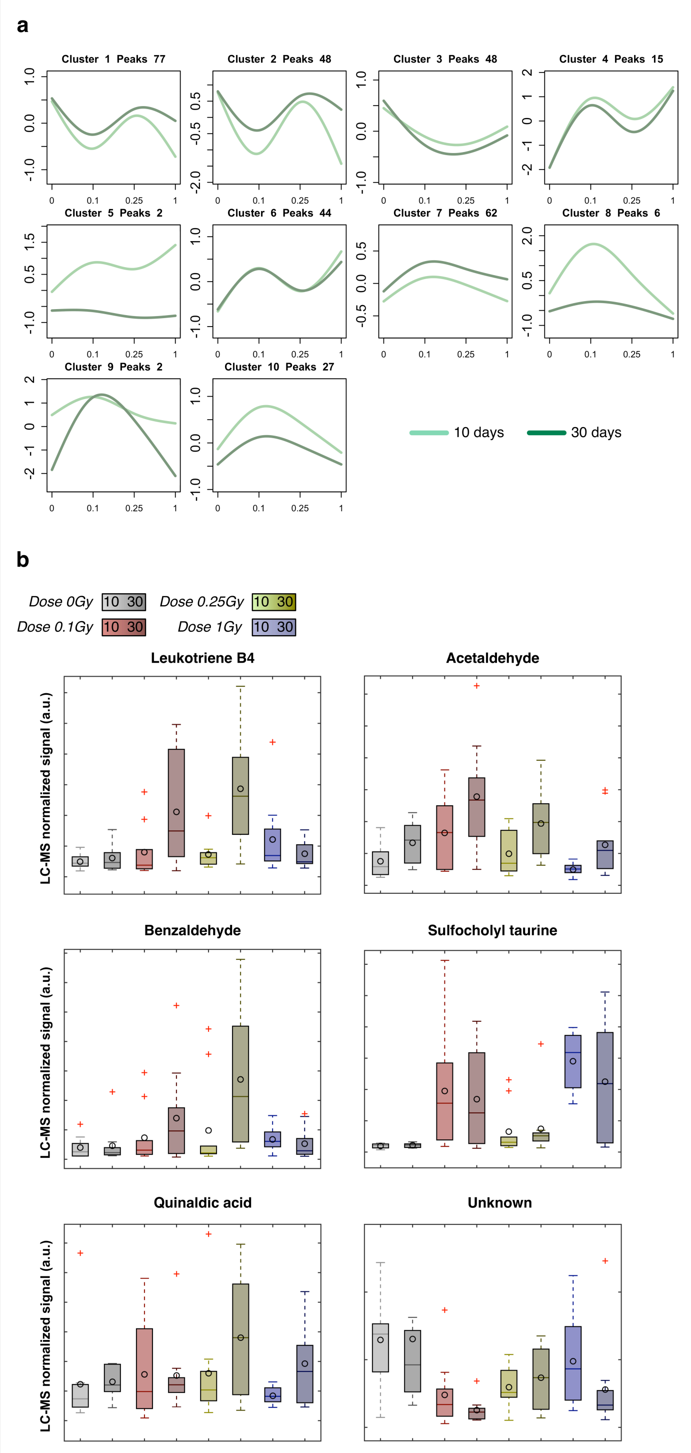

Figure S4: Metrics for Evaluation of Data Quality: Panels a, d: Overlay of total ion chromatograms of pooled quality controls samples showing minimal drifts in retention time across the batch acquisition. Panel b, c, e, f: Evaluation of mass accuracy using a standard mixture of metabolites (metmix) shows < 5 ppm error in ESI positive and negative modes.

a) ESI+, QC overlays

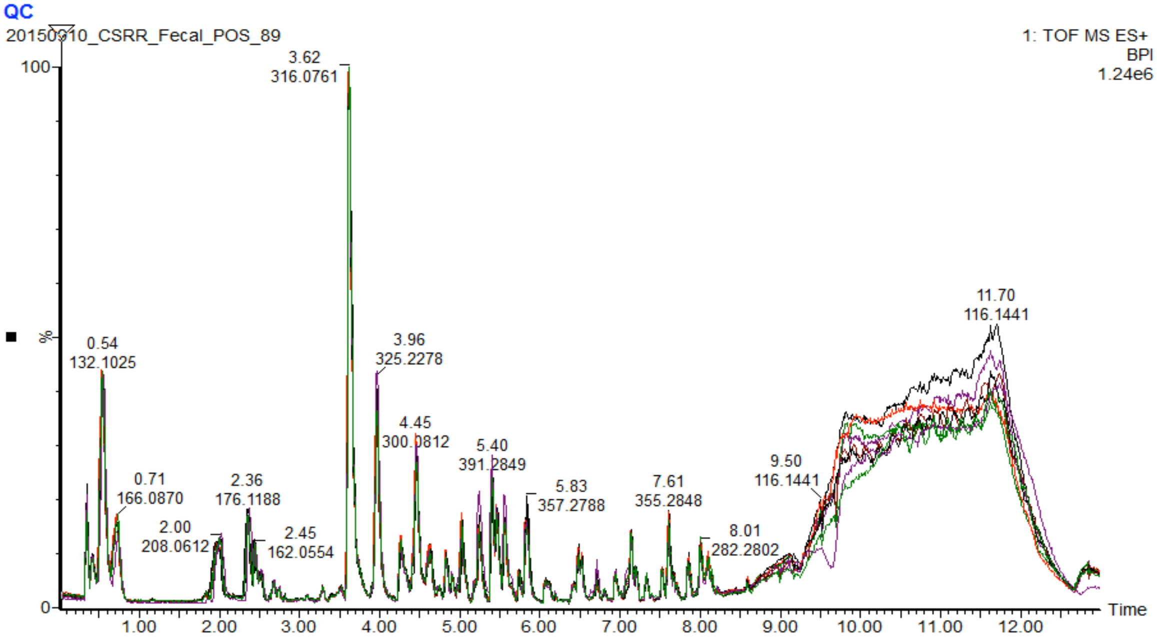

b) ESI+, before the run

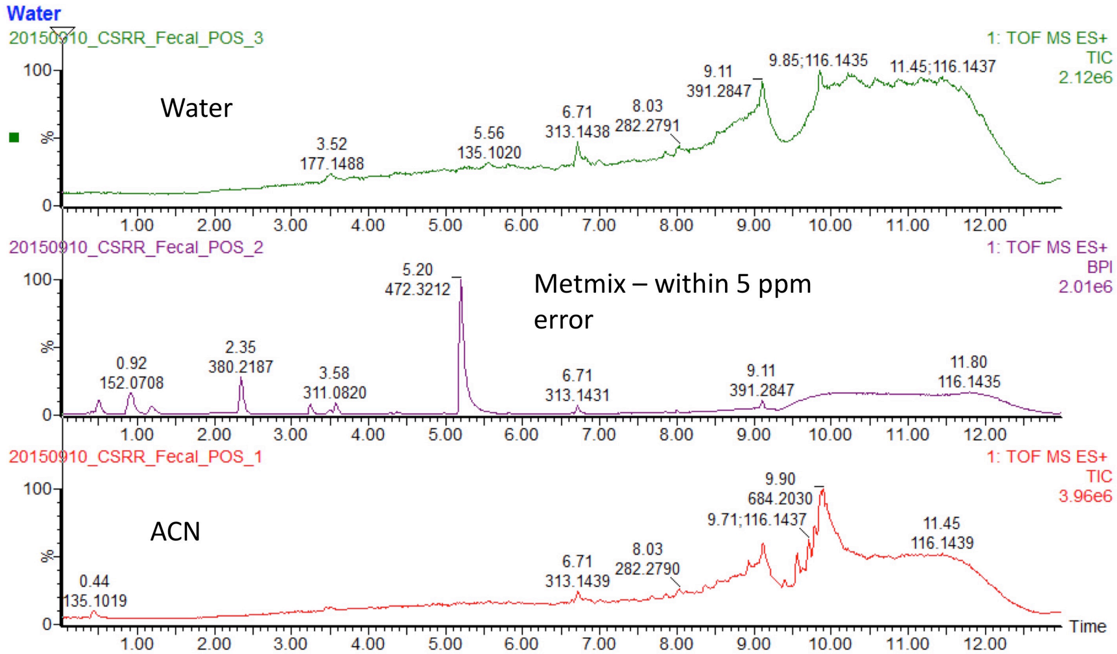

c) ESI+, after the run

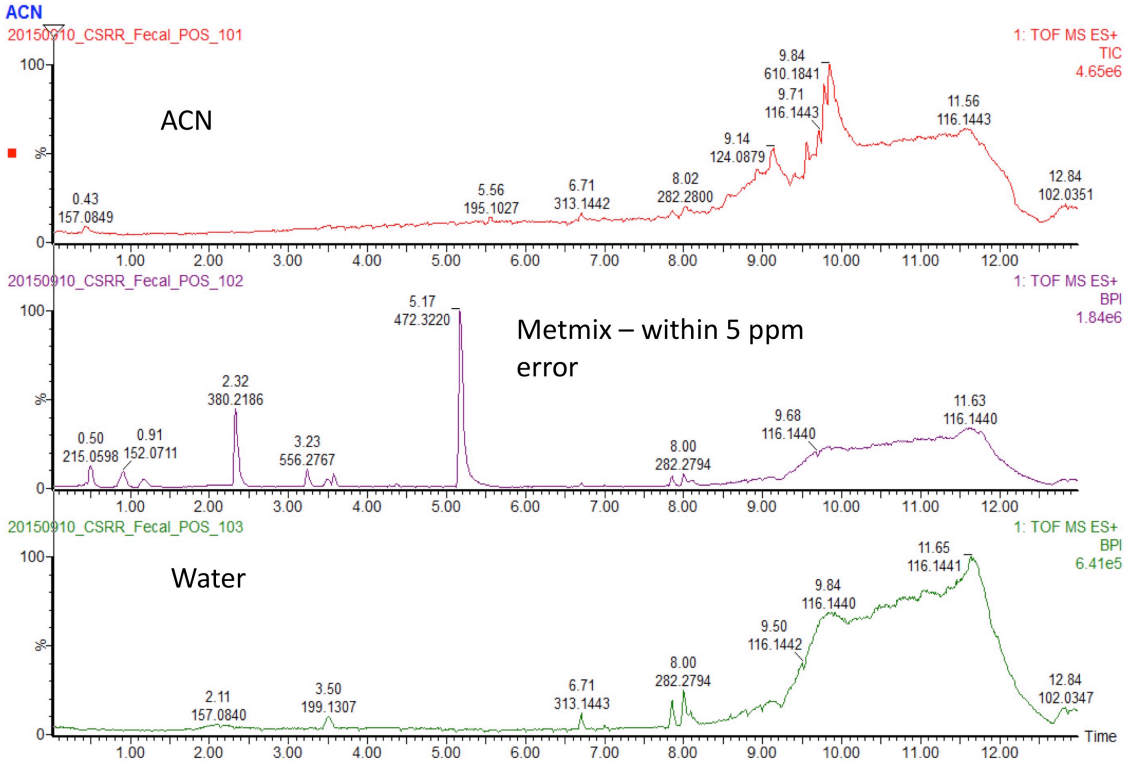

d) ESI-, QC overlays

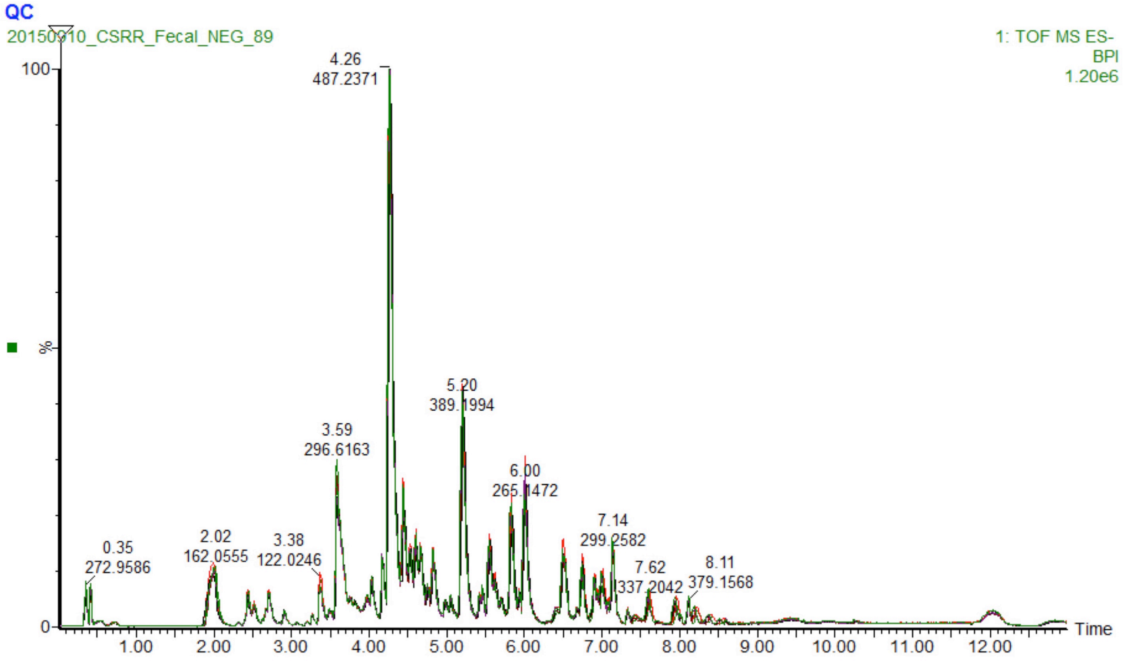

e) ESI-, before the run

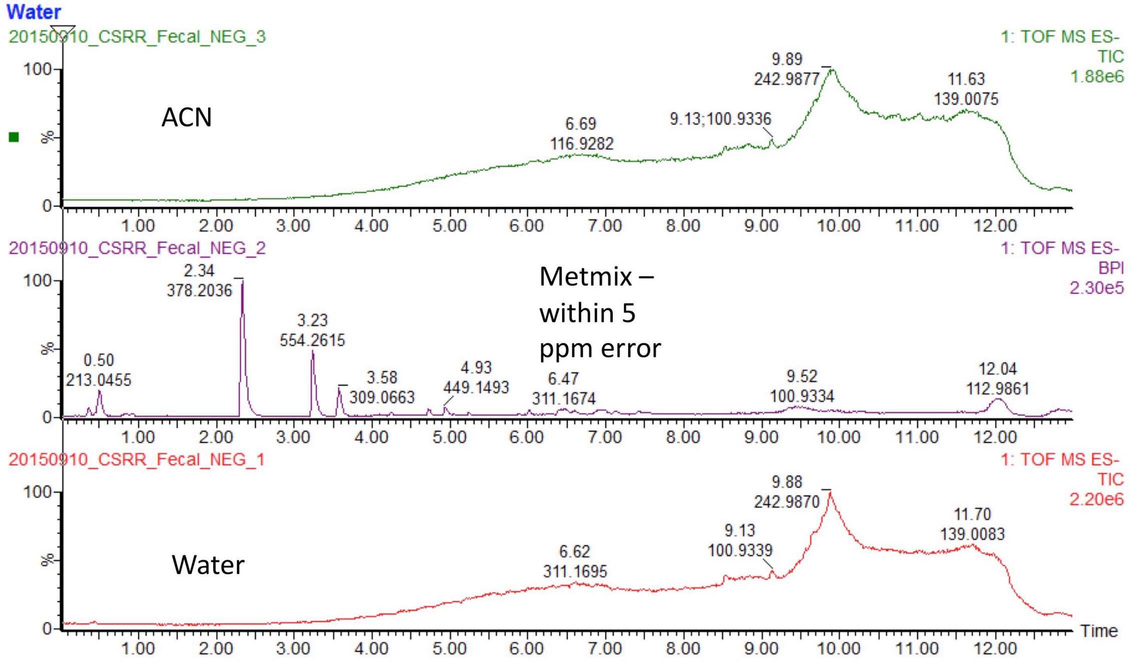

f) ESI-, after the run

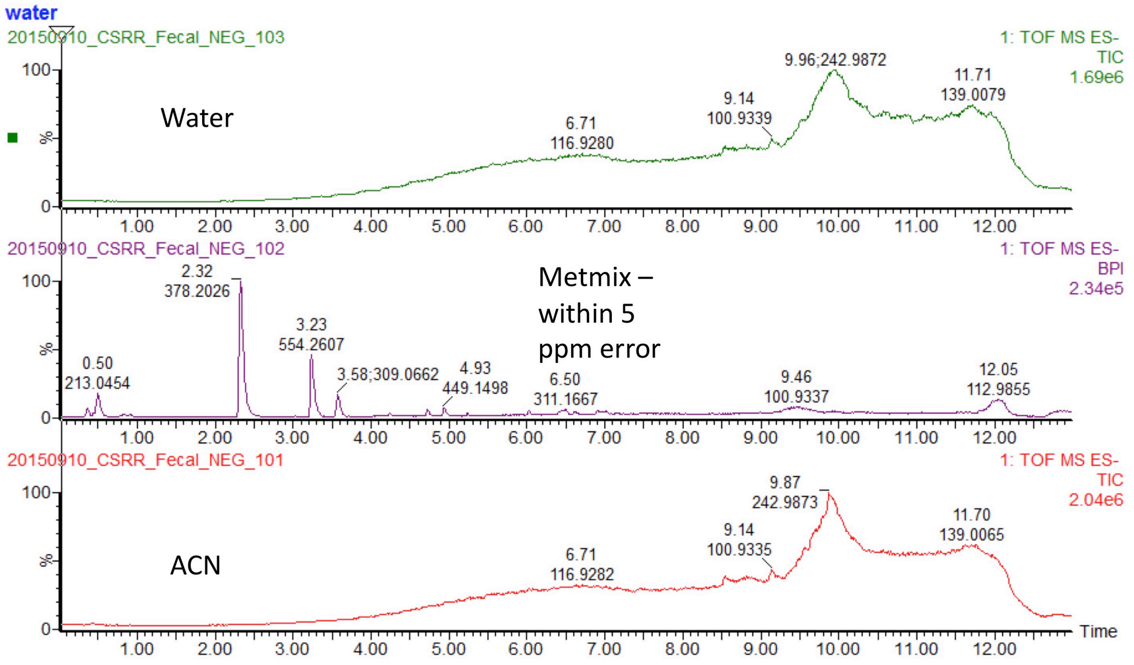

C. R preprocessing script, XCMS metabolomics tool

```
### Script modeled off of http://fiehnlab.ucdavis.edu/staff/kind/Metabolomics/Peak_Alignment/xcms/

### These are the 4 variables you have to set for your own datafiles anything else runs automatically

### Set your working directory under Windows, where your netCDF files are stored

### Organize samples in subdirectories according to their class names WT/GM, Sick/Healthy etc.

### Important: use "/" not "\". Make sure there is a "/" after the last folder in your 'myDir'

myDir = "C:/MassLynx/NetCDF files/TEST/"

myClass1 = "A"

myClass2 = "B"

myResultDir = "C:/MassLynx/NetCDF files/TEST/"

### change working directory to your files that you entered above, get working directory (make sure it matches what set before)

setwd(myDir)

 (WD <- getwd())

### load the xcms package

library(xcms)

### finds peaks in NetCDF

xset <- xcmsSet()

###  Group peaks together across samples

xset <- group(xset)

### calculate retention time deviations for every time and show fancy graphics

xset2<-retcor(xset,family="symmetric", plottype="mdevden")

###  Group peaks together across samples, set bandwitdh, change important m/z parameters here. Syntax: group(object, bw = 30, minfrac = 0.5, minsamp= 1,  mzwid = 0.25, max = 5, sleep = 0)

xset2<-group(xset2,bw=10)

###   identify peak groups and integrate samples. It is ok if you get warnings, it just means there will be some zero values in the data (less than with MarkerLynx, at least)

xset3<-fillPeaks(xset2)

### create report and save the result in EXCEL file, print 50 important peaks as PNG, include a metlin database search with 0.02 mass error

reporttab <- diffreport(xset3, myClass1, myClass2, myResultDir, 50, metlin = 0.02)

save(reporttab,file= "test.RData")

save.image(file = "test.RData")

write.csv(reporttab,"test.csv")
```
